# Toward automated classification of monolayer versus few-layer nanomaterials using texture analysis and neural networks

**DOI:** 10.1038/s41598-020-77705-8

**Published:** 2020-11-26

**Authors:** Shrouq H. Aleithan, Doaa Mahmoud-Ghoneim

**Affiliations:** Department of Physics, College of Science, King Faisal University, P. O. Box 400, Al-Ahsa, 31982 Kingdom of Saudi Arabia

**Keywords:** Materials science, Mathematics and computing, Nanoscience and technology, Physics

## Abstract

The need for a fast and robust method to characterize nanostructure thickness is growing due to the tremendous number of experiments and their associated applications. By automatically analyzing the microscopic image texture of MoS_2_ and WS_2_, it was possible to distinguish monolayer from few-layer nanostructures with high accuracy for both materials. Three methods of texture analysis (TA) were used: grey level histogram (GLH), grey levels co-occurrence matrix (GLCOM), and run-length matrix (RLM), which correspond to first, second, and higher-order statistical methods, respectively. The best discriminating features were automatically selected using the Fisher coefficient, for each method, and used as a base for classification. Two classifiers were used: artificial neural networks (ANN), and linear discriminant analysis (LDA). RLM with ANN was found to give high classification accuracy, which was 89% and 95% for MoS_2_ and WS_2_, respectively. The result of this work suggests that RLM, as a higher-order TA method, associated with an ANN classifier has a better ability to quantify and characterize the microscopic structure of nanolayers, and, therefore, categorize thickness to the proper class.

## Introduction

The rapid improvement and large number of publications related to two-dimensional materials, specifically two dimensional transition metals dichalcogenides (2D TMDs), reveal the exceptional properties of these materials that lead to novel applications in different areas. Their electronic, optical, optoelectronic, spintronic, and mechanical properties proved their candidacy for future lightweight, flexible, and more efficient devices^1–11^. Despite all the effort and progress in the work done related to this material, more work needs to be done in different aspects of growth before this material can enter the manufacturing line. One main goal is large area growth with homogeneous and high-quality material^12–15^. For that, detailed analysis needs to be done for each sample to examine the number of layers, defects, grain size, and doping which needs time and effort that can limit research and manufacturing progress.

TMDs are layered materials where each layer is composed of three atomic planes. The covalent bonds within one layer are strong compared to the van der Waals bond between layers, which allows for exfoliating the bulk crystal using different techniques to reach a mono to few-layer thickness. Exfoliation limits the size and location of the prepared samples, which is opposite to chemical vapor deposition (CVD) growth that has been used to grow large areas of high quality samples^16–18^. TMDs are well known for their indirect (inactive emission) to direct (active emission) bandgap transition as the material is thinned down from a bulk to a single layer structure^17,19^. The intense photoluminescence (PL) of monolayer structures compared to two-layer and thicker structures is an indication of the thickness of the sample^20,21^. Some Raman modes appear red or blue-shifted as the number of layers is increased^21,22^. The location, and in some materials like MoS_2_ the separation between two Raman modes, is taken as a fingerprint for the number of layers concerning different conditions of the sample like strain, doping, and defects that also affect the position of the Raman modes^19,21–24^. To determine the quality and the number of layers, samples need to be analyzed using PL, Raman, and other instruments to measure the direct thickness of the sample. Such analysis can limit the progress of the work toward large-area samples and their applications. Having a technique that can scan the sample optically to identify homogeneity in terms of the number of layers, defects, and grains can advance the progress toward full-chip device production.

Several attempts toward automated identification of layer number for 2D materials have been made in the literature. However, these attempts depend mainly on pixel optical intensity analysis which could fluctuate due to various conditions and, therefore, it does not represent a reliable method. Jessen et al. employed RGB (Red–Green–Blue) spectral fingerprints from all relevant parts of the light path—the light source, the detector, the two-dimensional film, and the substrate—to identify 2D materials. They also used grey level images; however, RGB retained more information for the analysis. The authors use various image processing methods to get a clear region or eliminate noise and refine the coverage using a combination of filters and algorithms. Some small features are removed in this process, and edges are also altered^25^. Masubuchia worked on a large set of SiO_2_/Si images where optical features were extracted then classified by a machine learning approach without the use of spectroscopic tools^26^. Xiaoyang Lin et al. applied a machine-learning strategy in the optical identification of 2D nanostructures, including graphene, MoS_2_, and their heterostructures. The machine learning optical identification method relies on trainable and automatic analyses of RGB optical information in photographs of 2D nanostructures^27^. Yuhao Li et al. used Fresnel law and machine learning methods to identify the number of layers in 2D materials. Optical contrast, RGB, and total color difference, were the features used in this work to simulate the visibility of 2D materials on Si/SiO_2_ substrate. The machine learning approach used was *k*-mean clustering. Images were reconstructed via RGB intensity and by distinguishing tiny color differences among layers^28^. Lei et al. reconstructed new images from raw optical ones that were acquired under arbitrary illuminants and cameras. The reconstructed images correspond to conditions of the specified illuminant and camera. Therefore, they were able to bypass the problem of condition variation and identify the layer number of 2D material. This work was applied to MoS_2_, WS_2_, and WSe_2_ layers^29^.

Image analysis in the literature tackling layer identification/count in 2D materials is relying so far on features extracted from images that are generally dependent on the optical intensity of pixels either grey levels or RGB. Although optical intensity is a rich source of information, this information is solely related to the independent pixels ignoring any relationships between pixels that could highlight an existing characteristic pattern, which makes it insufficient for the target standards of characterization required by industry. Moreover, optical intensity is highly dependent on acquisition conditions which require further processing to eliminate dependencies or continuously adjusting and fixing imaging conditions which are impractical, require time, and are more prone to errors. Since optical intensity analysis depends on the individual values of grey levels, it can also be sensitive to the dynamic range of grey levels at which the image is scaled, which shows another major drawback of this method. It becomes essential to investigate and develop more image analysis methods that dig deeper into the structures and pixel relationships to extract characteristic features for nanomaterials in general, and 2D TMDs in particular, and therefore assisting machine learning techniques and automatic characterization.

Texture Analysis (TA) is a well known and growing group of statistical and structural methods of image analysis that quantitatively evaluate pixels interdependent relationships and relate it to the characteristics of the underlying structure^30^. This technique was first applied by Haralick et al. on photomicrographic and satellite images to characterize different terrains and rocks^31^. The number and complexity of methods developed in TA have expanded since its first application, and more optimistic goals of characterization have been set and achieved, notably in the three dimensional analysis^32^. Each TA method produces numerous image features, and some, but not necessarily all, are unique for characterization.

Due to the robust findings of texture analysis, its application has been extended to different modalities of imaging, from photographic to medical and microscopic, with successful results in which some attempted to relate texture features to other quantitative variables^33–36^. The “order” of the TA method indicates the number of pixels involved in a single line of calculation. Texture is more precisely quantified by second order and higher-order methods. However, it is common to find in literature that pixel intensity features are also grouped with TA methods and referred to as first-order methods, which are mainly histogram parameters.

A prime advantage of TA is that it provides a non-destructive way of investigation that is reproducible, time-efficient, and can be applied to areas of different sizes, as a whole or partitioned, allowing wide-field characterization which is useful in industry. TA does not require the application of sophisticated filters to render structural features visible. This is because second and higher-order TA methods are less dependent on individual pixel values and more adapted to reveal subtle patterns that could be statistical or morphological. Grey level histogram (GLH), which calculates the probability distribution of grey level intensity is a first-order method, while co-occurrence matrix (COM), and run-length matrix (RLM) are second and higher-order methods, respectively. In this work, the three above mentioned methods are used and compared for accuracy introducing a future base for 2D TMD microscopic image analysis.

## Materials and methods

### Growth process

The TMD growth was performed using a two-inch tube furnace at atmospheric pressure. MoO_3_ (or WO_3_) powder was placed on a graphite holder with a mass of 5 mg. The substrate (Si with a layer of 100 nm or 300 SiO_2_ thickness) was placed face down on the same holder 1–2 cm away from the powder in the center of the furnace. The chalcogen was positioned just outside the furnace in a boron nitride boat. The furnace was ramped up to the growth temperature (780 °C for Mo based TMD growth and 850 °C for W-based TMD growth). As the furnace reached the growth temperature, the chalcogen was heated to 300 °C. A continuous flow of Argon was maintained at 50 sccm during the first 5 min temperature ramp, and the flow was then lowered to 10 sccm until the sample was removed. The growth time, which was typically between 10 to 15 min, was measured when the sulfur started to melt. At the end of growth time, the furnace was turned off and allowed to cool naturally back to room temperature, the above procedures of TMD growth were previously investigated by Aleithan (2018)^37,38^.

**Figure 1:**
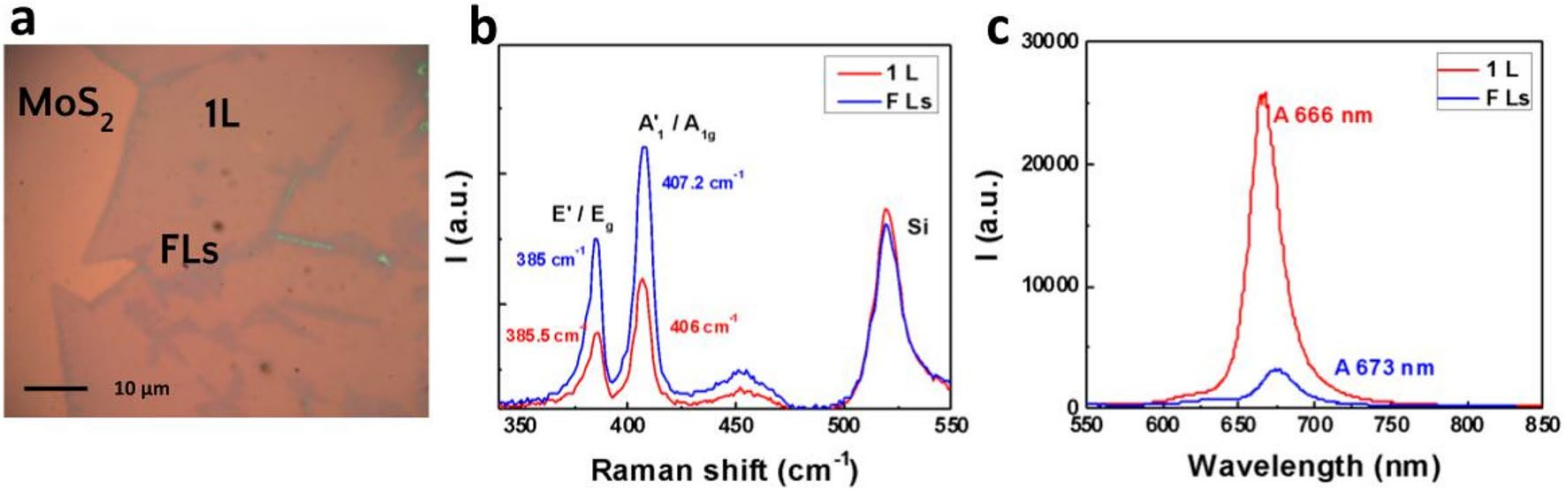
(**a**) White light optical image of a CVD grown monolayer (1L) and few-layer (FLs) MoS_2_ on Si/Sio_2_ substrate. (**b**) Raman signals for monolayer and few-layer positions [the labels (E^**`**^, A^**`**^_1_) are for 1L or an odd number of few-layers while (E_g_, A_1g_) are for an even number of few-layers^21^]. The location, and separation between the two Raman modes E_g_, and A_1g_ for each structure is an identification of the number of layers which directly relates to the sample preparation method. CVD grown samples can develop different levels of strain, doping, and defects that slightly affect the position of each Raman mode. (**c**) Photoluminescence signals for monolayer and few-layer positions with A excitonic peak that appears to be sharp and intense for 1L structure (direct, effective emission) compared to FLs (indirect, ineffective emission).

**Figure 2:**
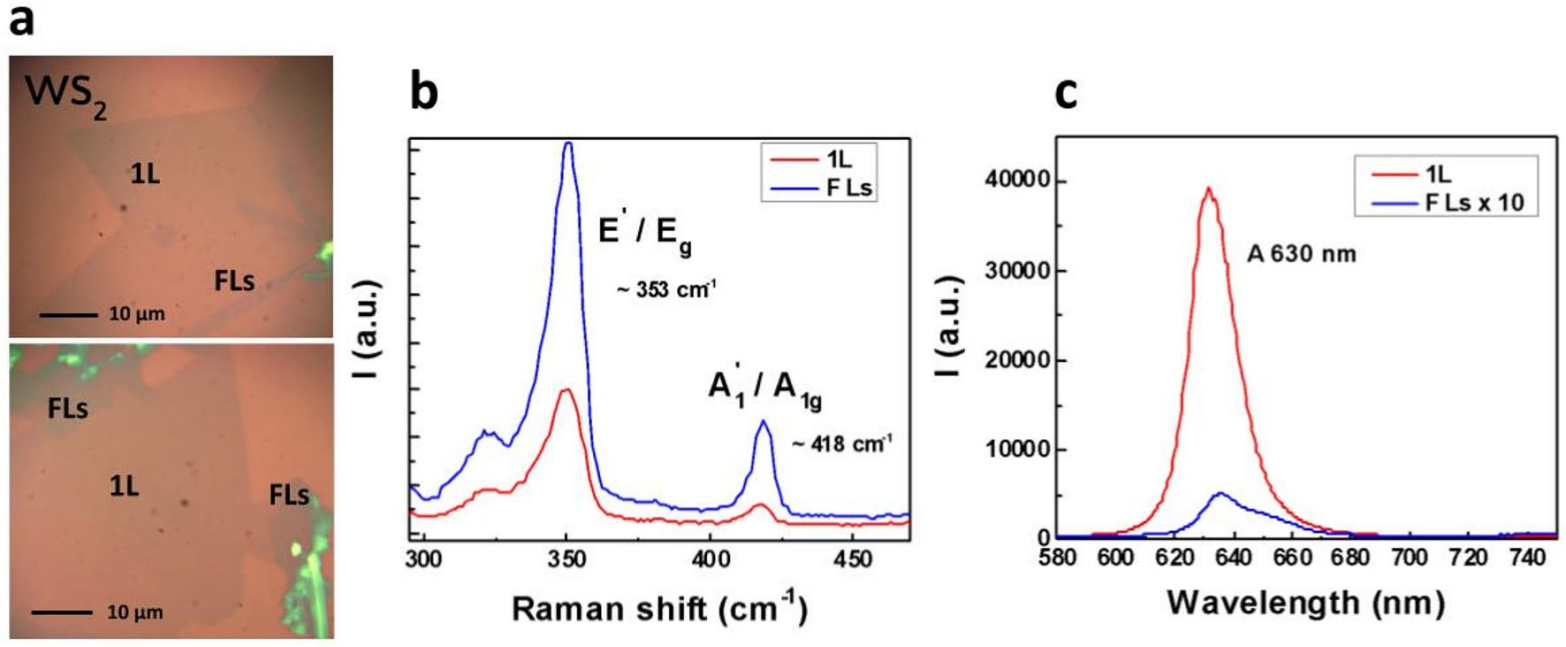
(**a**) White light optical image of a CVD grown monolayer (1L) and few-layer (FLs) WS_2_ on Si/SiO_2_ substrate. (**b**) Raman signals for monolayer and few-layer positions [(E^**`**^, A^`^_1_) for 1L, or an odd number of few-layers (E_g_, A_1g_) for an even number of few-layers ^21^]. (**c**) Photoluminescence signals for monolayer and few-layer positions with A excitonic peak that appear to be sharp and intense for 1L structure (direct, active emission) compared to FLs (indirect, inactive emission), which distinguish well between the two structures in case of WS_2_.

Optical images were collected using optical microscopy with white light illuminations and different objectives in terms of magnifications and numerical aperture (Figs. 1a and 2a). Raman and PL spectra were taken using a confocal microscope system (Witec) with 50X objective lens and 0.85 numerical aperture at room temperature with 0.9 mW, 532 nm, laser excitation (Figs. 1b,c and 2b,c). Several optical images for CVD grown MoS_2_ and WS_2_ on Si/SiO_2_ substrates have been analyzed using Raman and PL to identify monolayer and few-layer positions (Figs. 1, 2).

### Texture analysis

**Figure 3:**
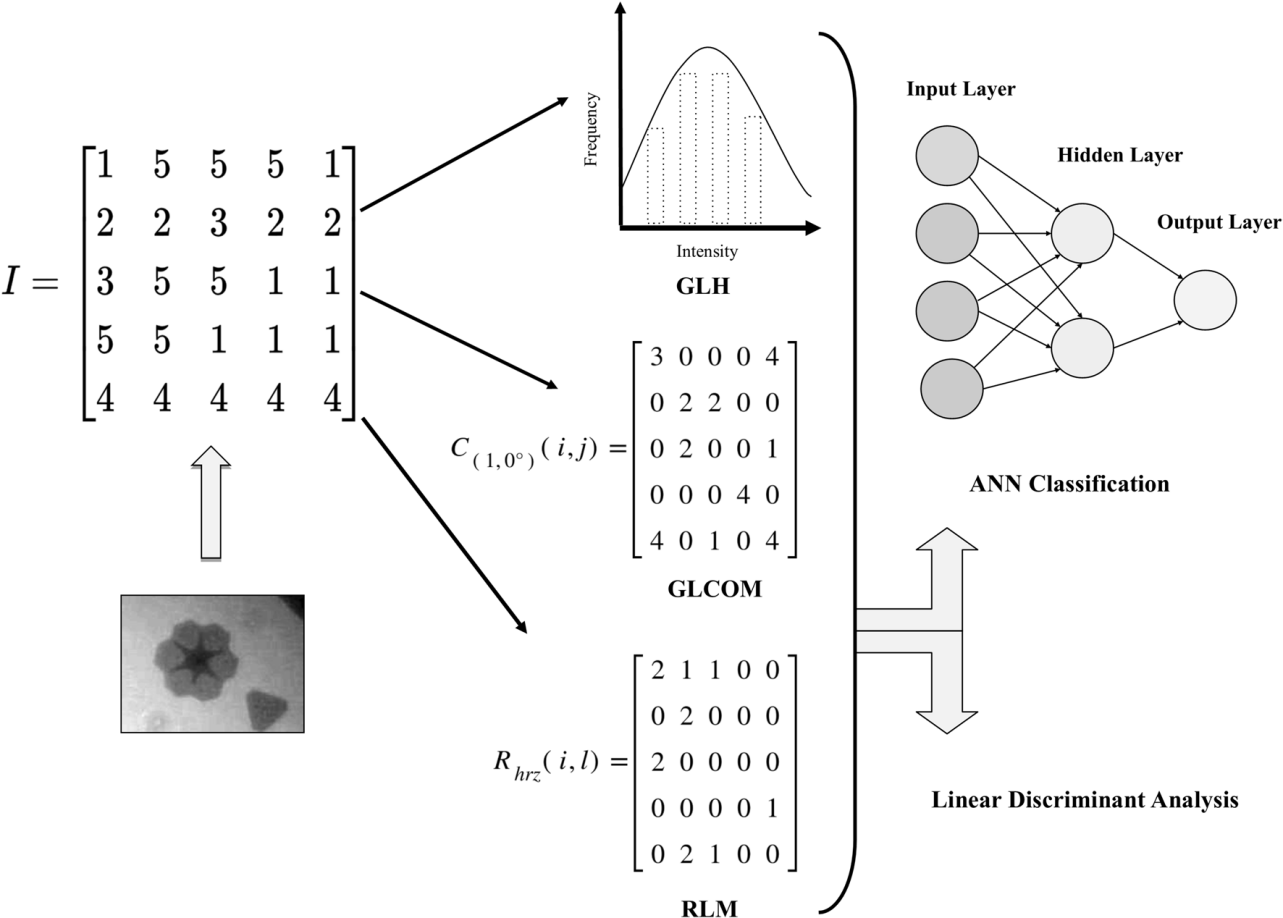
Texture analysis and feature classification processes starting from image *I*: the image in a form of a matrix (left column), texture features calculation (middle column) (using a first-order method: grey level histogram (GLH), second-order method: co-occurrence matrix (GLCOM) at distance *d* = 1 and angle *θ* = 0° *C*_*(*1,0°)_(*i*, *j*) where *i* and *j* are two grey levels in the image *I*, and higher-order method: run length matrix (RLM) calculated at the horizontal direction *R*_*hrz*_(*i*, *l*) where *i* is the grey level and *l* is the run, and finally classification using artificial neural network (ANN) or linear discriminant analysis (LDA) (right column). The ANN input layer corresponds to characterizing features from each method, and the ANN output layer is the classification results (monolayer class versus few-layer class). Note that *C*_*(*1,0°)_(*i*, *j*) and *R*_*hrz*_(*i*, *l*) are only two examples of the matrices calculated from GLCOM and RLM, respectively, on the image *I*. The ANN input layer or LDA input is composed of the best 10 (or 9 in case of GLH) features among all matrices combined in one method.

Three different statistical methods of TA have been applied to investigate the possibility of distinguishing between monolayer and few-layer nanostructures, those are grey level histograms (GLH), grey level co-occurrence matrix^31^ (GLCOM), and run-length matrix^31,39^ (RLM). Those methods are first-order, second-order, and higher-order methods, respectively. GLH measures the frequency of every grey level intensity in the image. Several statistics can be drawn from GLH such as mean, skewness, kurtosis, and percentiles. GLCOM, calculates a matrix *C*_*(d*, *θ)*_(*i*, *j*) of joint occurrence of two pixels of different or same grey level value that exist at a certain angle *θ* = (0°, 45°, 90°, or 135°) within a certain distance *d*, typically of (1, 2, 3, 4, or 5 pixels). Each GLCOM matrix is calculated for any given combination of *d* and *θ*, and usually for all combinations of the values above. From these matrices, many features can be calculated which mainly describe the homogeneity and correlation of texture. An example of how GLCOM is computed is given in Fig. 3. Consider *C*_*(*1,0°)_ (*i*, *j*) to be the GLCOM at distance *d* = 1 pixel and angle *θ* = 0°, each entry of the matrix (*i*, *j*) represents the number of times the two grey levels *i* and *j* occurred at the mentioned distance and angle in the image *I*. For example, the grey levels *i* = 5 and *j* = 1 (or *i* = 1 and *j* = 5) occurred four times at the above-mentioned conditions; therefore, the entry *C*_*(*1,0°)_(5,1) = *C*_*(*1,0°)_(1,5) = 4 in *C*_*(*1,0°)_ (*i*, *j*). Notice that for all entries *C*_*(*1,0°)_ (*i*, *j*) is equal to *C*_*(*1,0°)_ (*j, i*) which represents the sum of those two instances in reverse directions. This makes GLCOM a symmetric matrix about its diagonal. A more detailed explanation of GLCOM calculation and features can be found in literature^31^.

RLM calculates a matrix *R* in which a grey level *i* occurs with a run of length *l*. A run *l* is the number of consecutive pixels of the same value in the image at a certain direction. The matrix *R* is calculated in four directions: horizontal, vertical, 45°, and 135°. Each matrix produces several features that describe the image quantitatively. An example is given in Fig. 3, where a matrix R_*hrz*_(*i*,*l*) is calculated for all grey levels *i* that form horizontal runs *l* in the image *I*. The grey level *i* = 4 with run *l* = 5 in the horizontal direction happened once, therefore the entry R_*hrz*_(*4*,*5*) = 1 in the matrix (Fig. 3). A *coarse* texture is the one that is predominantly composed of long runs, while a *fine* texture is composed of short runs.

In this work, TA was done on 28 regions on MoS_2_ images (Fig. 1a) and 19 regions on WS_2_ images (Fig. 2a). Image equalization was done for all images to ensure the consistency of grey level dynamic range and to eliminate the effect of different conditions of brightness. Regions of analysis were delineated manually on each image by avoiding edges. Each region measured on average between 2000 and 4000 pixels.

The Fisher method of feature selection^40^ was used to automatically select and rank in ascending order the 10 most discriminant features among each of GLCOM and RLM parameters separately. Since GLH produced only 9 features, those were all used; however, these were also ranked according to their Fisher coefficient value (Tables 1, 2). An artificial neural network (ANN) classifier^41^ was used for classification, which takes the selected texture features as input layer and classifies image regions as output of two classes: monolayer or few-layer. The ANN classifier used in this work is a feedforward one that is composed of: an input layer of 10 nodes (or 9 in case of GLH, which represent features), a 1st hidden layer of 1 neuron, a 2nd hidden layer of 2 neurons, and an output layer of 2 nodes. ANN classifier learns to achieve the correct classification by adjusting synaptic weights between neurons in a process called backpropagation. The basic architecture of ANN can be demonstrated by a single layer ANN. A weight w is assigned to each input node which represents the strength of the node. Each synaptic weight (w_1_, w_2_, w_3_,…, w_n_) is then multiplied by the input feature (x_1_, x_2_, x_3_,…, x_n_), and then the sum (x_1_w_1_ + x_2_w_2_ + x_3_w_3_,… + x_n_w_n_) is calculated and fed into an activation function that works on assigning a class. Calculations become more sophisticated as the number of hidden layers increases. Linear discriminant analysis (LDA)^40,42^ is dimension reduction and classification method that is used as benchmark results. In LDA the between-class scatter and within-class scatter matrices are computed, then the eigenvalues and eigenvectors that maximize the ratio between the two are calculated. The same automatically selected features (9 for GLH, 10 for GLCOM, and 10 for RLM) were used as input for LDA. Figure 3 demonstrates a schematic representation of the characterization process. Results were compared between ANN and LDA (Table 3). Texture analysis, feature selection, ANN, and LDA classifications, were performed using MaZda-B11 software^43^ (version 4.5, 1999–2006) and MATLAB R2018b (1984–2018, The MathWork, Inc.).

**Table 1:** Characterizing features for MoS_2_ layers.

Parameter rank	First-order GLH	Second-order GLCOM	Higher-order RLM
1	Percentile 01%	Correlation (3, 135°)	Grey level non-uniformity 90°
2	Percentile 10%	Correlation (3, 45°)	Grey level non-uniformity 45°
3	Variance	Correlation (1, 45°)	Grey level non-uniformity 0°
4	Percentile 50%	Correlation (3, 90°)	Grey level non-uniformity 135°
5	Mean	Correlation (5, 90°)	Run length non-uniformity 0°
6	Skewness	Correlation (5, 135°)	Run length non-uniformity 45°
7	Percentile 90%	Correlation (1, 135°)	Run length non-uniformity 135°
8	Percentile 99%	SumVariance (1, 45°)	Run length non-uniformity 90°
9	Kurtosis	Contrast (3, 45°)	Long run emphasis 90°
10	–	Contrast (3, 135°)	Short run emphasis 0°

The best features, in descending order according to their Fisher coefficient rank, for characterizing MoS_2_ monolayer versus few-layer using first order: grey level histogram (GLH), second-order: grey level co-occurrence matrix (GLCOM) and higher-order: run-length matrix (RLM) methods. The brackets (*d*, *θ*) contain the values of distance and angle, respectively.

**Table 2:** Characterizing features for WS_2_ layers.

Parameter rank	First-order (GLH)	Second-order (GLCOM)	Higher-order (RLM)
1	Skewness	Difference variance (4, 45°)	Long run emphasis 90°
2	Percentile 01%	Contrast (3, 90°)	Long run emphasis 45°
3	Percentile 90%	Contrast (2, 90°)	Long run emphasis 0°
4	Mean	Difference variance (5, 45°)	Long run emphasis 135°
5	Percentile 99%	Contrast (4, 45°)	Grey level non-uniformity 0°
6	Percentile 50%	Difference variance (2, 90°)	Grey level non-uniformity 90°
7	Percentile 10%	Difference variance (3, 90°)	Grey level non-uniformity 135°
8	Kurtosis	Contrast (5, 45°)	Grey level non-uniformity 45°
9	Variance	Difference variance (4, 90°)	Fraction (of image in runs) 45°
10	–	Contrast (4, 90°)	Fraction (of image in runs) 135°

The best features, in descending order according to their Fisher coefficient rank, for characterizing WS_2_ monolayer versus few-layer using first order: grey level histogram (GLH), second-order: grey level co-occurrence matrix (GLCOM) and higher-order: run-length matrix (RLM) methods. The brackets (*d*, *θ*) contain the values of distance and angle, respectively.

**Table 3:** Classification results of MoS_2_ and WS_2_ layers.

Material	Classifier	GLH (%)	GLCOM (%)	RLM (%)
MoS_2_	ANN	79	79	89
	LDA	57	89	75
WS_2_	ANN	79	63	95
	LDA	79	79	68

Percentage accuracy of monolayer versus few-layer classification using two classifiers and three methods of texture analysis; First order: grey level histogram (GLH), second-order: grey level co-occurrence matrix (GLCOM) and higher-order: run-length matrix (RLM) methods, on Fisher selected features.

## Results and discussion

Among the three TA methods and the two different classifiers, RLM features combined with ANN classifier gave high classification accuracy (89% and 95% for MoS_2_ and WS_2_, respectively), while GLCOM with LDA gave high accuracy only for MoS_2_ (89%), which indicates that RLM features were superior in depicting the textural differences between monolayer and few-layer nanostructures. It can be shown that either for MoS_2_ or WS_2_, ANN was a better classifier using GLH and RLM input features (Table 3). In contrast, LDA was a better classifier using GLCOM input features. These results suggest that RLM features, as a higher-order method, could be more reliable in the learning process that is used in neural networks. The association of texture parameters with structural properties is still an unexplored area in literature. However, it is systematically known that it requires a cognitive effort to visually distinguish between two textures that are similar in their second order texture characteristics (or moments) but different at a higher-order^44^. Which means that features of RLM, as a higher-order method, are more in-depth descriptors of the underlying texture. Tables 1 and 2, show a list of GLCOM discriminating features which by definition are measures of the homogeneity of grey level distribution (such as correlation, contrast, …etc.). On the other hand, it can be demonstrated that RLM discriminating features (Tables 1, 2) are those that reflect texture granularity through estimating “run” properties. In association with classification results, this could suggest that granularity is generally a better characterizing property than homogeneity for automated classification of nanolayers thickness. In addition, this characterization requires a higher-order calculation for achieving higher accuracy for some materials such as in WS_2_ (Table 3). The ability of the classifier to categorize samples based on these features depends on the mathematical model it uses and its ability to emphasize class separability with given input features. This might explain why one classifier works better than the other with some texture features: in the current findings, ANN with RLM features, and contrarily, LDA with GLCOM features, in the cases of both MoS_2_ and WS_2_ (Table 3).

TMDs are layered materials, where each layer has a total thickness of 6 to 7 Å. Each layer is composed of three atomic planes: hexagonally packed metal atoms sandwiched between two planes of chalcogen atoms. The covalent intralayer bonds (Mo-S) are strong compared to the weak interlayer van der Waals bonds. For monolayer structures, one layer of materials (7 Å thin layer) lies on Si/SiO_2_ substrate while for a few-layer structure, up to 5 layers of materials (thickness < 35 Å) lie on the substrate. That change in thickness plays the main role in the color contrast that is observed in the optical images between monolayer and few-layer structures, which is a thin-film interference-effect. Interference effects change pixel grey level intensity which directly affects the first-order method (GLH) features. For example, the “Mean” value represents the average pixel optical intensity on a selected region. This value becomes a discriminating feature when it is statistically consistent among one class (monolayer or few-layer), and so on for the rest of GLH features. GLH features can be misleading if illumination conditions are not consistent, or pre-processing images were not equalized in a way to remove light inhomogeneity, besides, using different thicknesses of SiO_2_ or different substrates, which could be challenging in a day to day laboratory practice. On the molecular structural level, MoS_2_ has hexagonally repeating patterns, and similarly WS_2_. Both compounds have crystal lamellar structures. At this stage it cannot be confirmed that the second or higher-order methods can detect the details of this structure or correlate with its patterns; however, it can be argued that the repeating patterns of both compounds played a significant role in the superior results of RLM as a higher-order method.

Knowing the structural properties of the underlying nanostructures and the problem in hand, can both help in selecting the TA method and classifier which are expected to work best for a certain categorization task, and, therefore, avoid time-consuming trial and error or computationally extensive random application of numerous methods.

## Conclusion

Monolayer versus few-layer characterization has been achieved by analyzing the microscopic image texture of MoS_2_ and WS_2_. The classification accuracy is high for both materials. Three methods of texture analysis (TA) were used: grey level histogram (GLH), grey levels co-occurrence matrix (GLCOM), and run-length matrix (RLM), which correspond to first, second, and higher-order statistical methods, respectively. Two classifiers were used: artificial neural networks (ANN), and linear discriminant analysis (LDA), on the best automatically selected features for each method. RLM with ANN has given the highest percentage accuracy of classification, which was 89% for MoS_2_ and 95% for WS_2_, respectively. GLCOM with LDA has given high accuracy only for MoS_2_. GLH cannot be a reliable method for nanolayer thickness characterization at its results were less accurate. Also, because GLH describe characteristics that can be easily altered by surrounding experimental conditions. According to this result, RLM is suggested to be a better method to quantify the microscopic structure of nanolayers associated with its thickness, and, therefore, assigning thickness to the proper class.

Texture analysis associated with ANN opens doors for fast automated and accurate characterization of small or large area grown 2D TMDs using optical microscopy. The optical characteristics of the 2D materials are playing key roles in device manufacturing and controlling performance. A better understanding of the rules that control the automated characterization of nanolayers using texture features is going to be achieved when the physical and chemical optical properties can be properly related to the formation of underlying structures, which could be a rich subject for future work.

## Data Availability

The datasets generated during and/or analyzed during the current study are available from the corresponding author upon reasonable request.
